# Inferior vena cava diameters and collapsibility index changes reveal early volume depletion in a healthy donor model

**DOI:** 10.1186/2036-7902-4-S1-A29

**Published:** 2012-12-18

**Authors:** Paolo Pasquero, S Albani, E Sitia, F Castagno, S D’Antico, M Porta

**Affiliations:** 1 Department of Medicine, S. Giovanni Battista Hospital, Turin, Italy; 2 Blood Bank, S. Giovanni Battista Hospital, Turin, Italy

## Background

Measurement of the Inferior Vena Cava (IVC) diameters and collapsibility index (CI) for the detection of early volume depletion in healthy donors was recently investigated by Resnick et al. who showed no significant changes using different approaches after blood loss.

## Objective

To investigate the usefulness of IVC diameters and CI measurement to detect early volume depletion after blood loss of 400-450 ml using different sonographic windows.

## Patients and methods

54 consecutive healthy donors were enrolled. 7 were excluded (1 for anaemia, 1 hypotension, 4 inadequate IVC visualization and 1 abnormal IVC dilatation).

Ultrasound measurements were obtained using M Mode, immediately before and as soon as possible after blood donation. Time from end of donation was recorded. Antero-posterior mid hepatic IVC diameter (long-axis and short-axis) were taken under the hepatic veins while distal IVC diameter was measured at the left renal vein junction. All data were fully recorded for a post processing study with an open source software (Image J).

## Results

47 donors (27 males, median age 38, median BMI 24.25) had satisfactory IVC visualization in 92.1%, 88.2%, 58.8% of cases for hepatic long-axis, hepatic short-axis and renal vein approach respectively.

No statistical difference between real time and post processing hepatic short-axis measurements was found. All mean IVC diameters decreased (-19.3%, -19.8%, -25.9% in maximal diameters; -30.3%, -32.4%, -34.7% in minimal diameters) and mean CI increased (+27.2%,+30.2%, +23.9%) for each window after blood letting (p<0.00). Mid hepatic IVC long-axis revealed a correlation between the time after donation and CI ( 20% CI decrease within 5 minutes until 0% after 20 minutes).

## Conclusion

Both IVC diameters and CI changes identified volume depletion in a healthy donor model. The mid hepatic long-axis window showed the best correlation between the IVC-CI and early volume variations following blood loss and post-donation volume repletion.

## References

[B1] Resnick J Cydulka R Platz E Jones R J Emerg Med 2011 41 3 270 5 10.1016/j.jemermed.2010.11.040 21421294

